# Quantum Games with Unawareness

**DOI:** 10.3390/e20080555

**Published:** 2018-07-26

**Authors:** Piotr Frąckiewicz

**Affiliations:** Institute of Mathematics, Pomeranian University, 76-200 Slupsk, Poland; fracor6@icloud.com

**Keywords:** quantum game, game with unawareness, strategic-form game, quantum algorithms

## Abstract

Games with unawareness model strategic situations in which players’ perceptions about the game are limited. They take into account the fact that the players may be unaware of some of the strategies available to them or their opponents as well as the players may have a restricted view about the number of players participating in the game. The aim of the paper is to introduce this notion into theory of quantum games. We focus on games in strategic form and Eisert–Wilkens–Lewenstein type quantum games. It is shown that limiting a player’s perception in the game enriches the structure of the quantum game substantially and allows the players to obtain results that are unattainable when the game is played in a quantum way by means of previously used methods.

## 1. Introduction

Game theory, launched in 1928 by John von Neumann in a paper [1] and developed in 1944 by John von Neumann and Oskar Morgenstern in a book [2] is one of the youngest branches of mathematics. The aim of this theory is mathematical modeling of behavior of rational participants of conflict situations who aim at maximizing their own gain and take into account all possible ways of behaving of remaining participants. Within this young theory, new ideas that improve already used models of conflict situations are still proposed. One of the latest trends is to study games with unawareness, i.e., games that describe situations in which players behave according to his own view of the game, and consider how all the remaining players view the game. This way of describing of a conflict situation goes beyond the most frequently used paradigm, according to which it is assumed that all participants in a game have full knowledge of the situation.

The other, and even younger, field developed on the border of game theory and quantum information theory is quantum game theory. This is an interdisciplinary area of research within which considered games are supposed to be played with the aid of objects that behave according to the laws of quantum mechanics, and in which non-classical features of these objects are relevant to the way of playing and results of a game.

Seventeen years of research on quantum game theory that began with Reference [3] has been conducted within two main streams. On the one hand, the schemes of playing quantum games were defined with the aid of notions and methods of quantum information (quantum noise, quantum random walking, superquantum operations, etc.). On the other hand, quantum game theory is formed on the basis of classical game theory, i.e., within quantum game theory are studied such problems as refinements of Nash equilibria (e.g., evolutionarily stable strategies [4,5,6,7] or correlated equilibria [8,9,10]), games in extensive form [3,11,12], repeated games [13,14] as well as elements of cooperative games theory [15,16]. Our work may be placed within this stream. It aims to introduce to the theory of quantum games the idea of games with unawareness.

The basic object studied in game theory and quantum game theory is a game in strategic form. Actually, this is a very simplified model of reality, since it assumes that each of the players knows the exact form of the game, in particular that every player knows the sets of strategies of all remaining players. Moreover, every player is aware that the remaining players know the exact form of the game. Further, every Player *i* knows that the remaining players know that the Player *i* knows the exact form of the game, etc. In other words, it is assumed that the knowledge of the game is common knowledge among the players. This is quite a strong assumption with respect to modeling real-world situations, since it is rather hard to assume that, in a real decision problem, every player has complete knowledge about all possible actions of the remaining players, or he has complete knowledge of how the remaining players value their strategies, i.e., that he knows their payoffs functions completely. Finally, it cannot be sure that he knows precisely how many players take part in a decision problem. In reality, various players may have different perceptions of a game. Moreover, they may have different perceptions of how other players view the game. As a result, a game originally described as a single strategic-form game, when modeled in a real world may require more precise description and should be replaced by a family of games in which each game corresponds to perception of a particular player.

In our opinion, quantum games with unawareness are natural generalization of up to now developed theory of quantum games. In this paper, we extend a quantum game to a model that specifies how each player views the game (whether it is classical or quantum), how she views the other players view the game, and so on. With this setup, we examine the rational results of the game under the notion of extended Nash equilibrium—the solution concept defined for games with unawareness.

## 2. Preliminaries on Games with Unawareness

This section is based on [17]. We review relevant material for the notion of strategic-form games with unawareness. The reader who is not familiar with this topic is encouraged to follow the definitions together with the introductory example below. The paper [18] provides the reader with similar preliminaries on games with unawareness

### 2.1. Introductory Example

Suppose two players play the following bimatrix game:

(1) $$G_{\emptyset }=\begin{array}{cc} & \begin{array}{ccc} b_{1}\; & \;b_{2}\; & \;b_{3} \end{array}\\ \begin{array}{c} a_{1}\\ a_{2}\\ a_{3} \end{array} & \left(\begin{array}{ccc} \left(2,2\right) & \left(1,1\right) & \left(2,2\right)\\ \left(3,3\right) & \left(1,2\right) & \left(1,4\right)\\ \left(4,1\right) & \left(2,2\right) & \left(0,0\right) \end{array}\right) \end{array}.$$

Let us consider the case that Players 1 and 2 are not aware of the third strategy of their opponent. That is, Player 1 views the game in the form

(2) $$G_{1}=\begin{array}{cc} & \begin{array}{cc} b_{1}\; & \;b_{2} \end{array}\\ \begin{array}{c} a_{1}\\ a_{2}\\ a_{3} \end{array} & \left(\begin{array}{cc} \left(2,2\right) & \left(1,1\right)\\ \left(3,3\right) & \left(1,2\right)\\ \left(4,1\right) & \left(2,2\right) \end{array}\right) \end{array}.$$

whereas Player 2 perceives that the game is of the form

(3) $$G_{2}=\begin{array}{cc} & \begin{array}{ccc} b_{1}\; & \;b_{2}\; & \;b_{3} \end{array}\\ \begin{array}{c} a_{1}\\ a_{2} \end{array} & \left(\begin{array}{ccc} \left(2,2\right) & \left(1,1\right) & \left(2,2\right)\\ \left(3,3\right) & \left(1,2\right) & \left(1,4\right) \end{array}\right) \end{array}.$$

Further, suppose that each player finds that their third strategy is hidden from the opponent. In other words, each player finds that the other player is considering the following game:

(4) $$G_{12}=G_{21}=\begin{array}{cc} & \begin{array}{cc} b_{1}\; & \;b_{2} \end{array}\\ \begin{array}{c} a_{1}\\ a_{2} \end{array} & \left(\begin{array}{cc} \left(2,2\right) & \left(1,1\right)\\ \left(3,3\right) & \left(1,2\right) \end{array}\right) \end{array}.$$

Moreover, the higher order views (for example, the view v=121 that Player 1 finds that Player 2 finds that Player 1 is considering) are associated with Equation (4), i.e., $G_{v}=G_{12}=G_{21}$ for v∈{121,212,1212,2121,…}.

The problem presented above is an example of a strategic-form game with unawareness that can be formally described by a family of games ${\left\{G_{v}\right\}}_{v\in N\cup \{\emptyset \}}$ where N={1,2,12,21,121,212,…}. The set N∪{∅} (with typical element *v*) consists of the relevant views. The view v=∅ corresponds to the modeler’s game—the actual game played by the players. In our example, this is the game in Equation (1).

A basic solution concept is a Nash equilibrium (see Definition 2). One can check that the game in Equation (1) has the unique equilibrium payoff profile (2,2) generated, for example, by the strategy profile $\left(a_{3},b_{2}\right)$. Although both players are aware of their own strategies, they are not aware of the whole game in Equation (1), and, apart from that, they perceive the game seen by the other player in different ways. Thus, it is not evident that the game ends with outcome (2, 2). Since Player 1 finds that Player 2 perceives the game in Equation (4), she may deduce that Player 2 plays according to the unique Nash equilibrium $\left(a_{2},b_{1}\right)$ of Equation (4). Therefore, Player 1’s best reply to $b_{1}$ is $a_{3}$ as she perceives Equation (2).

In the same manner, Player 2 deduces that Player 1 plays the equilibrium strategy $a_{2}$ in Equation (4). Since Player 2 views the game in Equation (3), his best reply to $a_{2}$ is $b_{3}$. As a result, the strategy profile $\left(a_{3},b_{3}\right)$ with the worst possible payoffs for the players is predicted to be a reasonable outcome in the game ${\left\{G_{v}\right\}}_{v\in N\cup \{\emptyset \}}$ described by Equations (1)–(4).

The game result $\left(a_{3},b_{3}\right)$ can be directly determined by the extended Nash equilibrium [17]—a counterpart of the notion of Nash equilibrium in games with unawareness. The formal definition is presented in Section 2.3. Here, we simply provide the result of applying the extended Nash equilibrium to Equations (1)–(4). The unique equilibrium is a family of strategy profiles ${\left\{{\left(\sigma \right)}_{v}\right\}}_{v\in N\cup \{\emptyset \}}$ defined as follows:

(5) $$\sigma_{v}=\left\{\begin{array}{cc} \left(a_{3},b_{3}\right) & \mathrm{if}\;v=\emptyset ,\\ \left(a_{3},b_{1}\right) & \mathrm{if}\;v=1,\\ \left(a_{2},b_{3}\right) & \mathrm{if}\;v=2,\\ \left(a_{2},b_{1}\right) & \mathrm{otherwise}. \end{array}\right.$$

The profiles in Equation (5) coincide with the reasoning we already used to determine the outcome $\left(a_{3},b_{3}\right)$. The result of the game corresponds to the modeler’s view v=∅. The result $\left(a_{3},b_{1}\right)$ seen from Player 1’s point of view corresponds to the view v=1. Player 2 predicts the outcome $\left(a_{2},b_{3}\right)$ corresponding to the view v=2.

### 2.2. Strategic-Form Games with Unawareness

Let $G=(N,{\left(S_{i}\right)}_{i\in N},{\left(u_{i}\right)}_{i\in N})$ be a strategic form game. This is the game considered by the modeler. Each player may not be aware of the full description of *G*. Hence, $G_{v}=\left(N_{v},{\left({\left(S_{i}\right)}_{v}\right)}_{i\in N_{v}},{\left({\left(u_{i}\right)}_{v}\right)}_{i\in N_{v}}\right)$ denotes Player v’s view of the game for v∈N. That is, the Player v views the set of players, the sets of players’ strategies, and the payoff functions as $N_{v}$, ${\left(S_{i}\right)}_{v}$ and ${\left(u_{i}\right)}_{v}$, respectively. In general, each player also considers how each of the other players views the game. Formally, with a finite sequence of players $v=(i_{1},\ldots ,i_{n})$, there is associated a game $G_{v}=\left(N_{v},{\left({\left(S_{i}\right)}_{v}\right)}_{i\in N_{v}},{\left({\left(u_{i}\right)}_{v}\right)}_{i\in N_{v}}\right)$. This is the game that Player $i_{1}$ considers that Player $i_{2}$ considers that …Player $i_{n}$ is considering. A sequence *v* is called a view. The empty sequence v=∅ is assumed to be the modeler’s view, i.e., $G_{\emptyset }=G$. We denote an action profile $\prod_{i\in N_{v}}s_{i}$ in $G_{v}$, where $s_{i}\in {\left(S_{i}\right)}_{v}$ by ${\left(s\right)}_{v}$. The concatenation of two views $\bar{v}=\left(i_{1},\ldots ,i_{n}\right)$ followed by $\tilde{v}=\left(j_{1},\ldots ,j_{n}\right)$ is defined to be v=v¯^v˜=(i1,…,in,j1,…,jn). The set of all potential views is $V={⋃}_{n=0}^{\infty }N^{\left(n\right)}$ where $N^{\left(n\right)}=\prod_{j=1}^{n}N$ and $N^{\left(0\right)}=\emptyset$.

**Definition** **1.** *A collection ${\left\{G_{v}\right\}}_{v\in V}$ where V⊂V is a collection of finite sequences of players is called a strategic-form game with unawareness and the collection of views V is called its set of relevant views if the following properties are satisfied:*

*1.*  *For every v∈V,*
  (6) v^v∈Vif and only ifv∈Nv.
*2.*  *For every v^v˜∈V,*
  (7) v∈V,∅≠Nv^v˜⊂Nv,∅≠(Si)v^v˜⊂(Si)vfor alli∈Nv^v˜.
*3.*  *If v^v^v¯∈V, then*
  (8) v^v^v^v¯∈VandGv^v^v¯=Gv^v^v^v¯.
*4.*  *For every strategy profile (s)v^v˜={sj}j∈Nv^v˜, there exists a completion to a strategy profile (s)v={sj,sk}j∈Nv^v˜,k∈Nv\Nv^v˜ such that*
  (9) (ui)v^v˜((s)v^v˜)=(ui)v((s)v).

The first condition says what views are, in fact, relevant. If, for example, the set of players $N_{1}$ perceived by Player 1 does not contain, say, Player 3, i.e., $3\notin N_{1}$, the view 1^3 what Player 1 thinks that Player 3 is considering is not relevant to strategic position of Player 1. Therefore, 1^3∈V.

The second condition, in particular, states that if, Player 1 finds that Player 2 is considering a player or a strategy as a part of the game, he himself considers those elements in the game he perceives.

The third condition requires that if a player finds a game $G_{1}$, he also finds that he has that perception, i.e., G1^1=G1. More generally, if Player 1 finds that Player 2 finds that Player 1 is considering a given game, then Player 1 is aware that Player 2 knows that he finds that Player 1 is considering that game.

Games $G_{v}$ corresponding to some views and the modeler’s game $G_{\emptyset }$ may differ with respect to the number of players. Since the payoffs are the result of strategies chosen by all the players, the payoffs in a restricted game $G_{v}$ (with possibly a smaller number of players) may not be uniquely determined. The fourth condition says that the payoffs in a restricted game are the payoffs in the game with more players by adding some strategy profile of these players. In other words, a restricted game does not contain new payoffs.

### 2.3. Extended Nash Equilibrium

A basic solution concept for predicting players’ behavior is a Nash equilibrium [19].

**Definition** **2.** *A strategy profile $s^{*}=\left(s_{1},s_{2},\ldots ,s_{n}\right)$ is a Nash equilibrium if for each Player i∈{1,…,n} and each strategy $s_{i}$ of Player i*

(10) $$u_{i}\left(s^{*}\right)\geq u_{i}\left(s_{i},s_{-i}^{*}\right),$$

*where $s_{-i}^{*}:={\left(s_{j}\right)}_{j\neq i}$.*

To define the Nash-type equilibrium for a strategic-form game with unawareness, it is needed to redefine the notion of strategy profile.

**Definition** **3.** *Let ${\left\{G_{v}\right\}}_{v\in V}$ be a strategic-form game with unawareness. An extended strategy profile (ESP) in this game is a collection of (pure or mixed) strategy profiles ${\left\{{\left(\sigma \right)}_{v}\right\}}_{v\in V}$, where ${\left(\sigma \right)}_{v}$ is a strategy profile in the game $G_{v}$ such that for every v^v^v¯∈V holds*

(11) (σv)v=(σv)v^vas well as(σ)v^v^v¯=(σ)v^v^v^v¯.

To illustrate Equation (11), let us take the game $G_{12}$—the game that Player 1 thinks that Player 2 is considering. If Player 1 assumes that Player 2 plays strategy ${\left(\sigma_{2}\right)}_{12}$ in the game $G_{12}$, she must assume the same strategy in the game $G_{1}$ that she considers, i.e., ${\left(\sigma_{2}\right)}_{1}={\left(\sigma_{2}\right)}_{12}$. In our introductory example, Player 1 finds that Player 2 is considering strategy ${\left(\sigma_{2}\right)}_{12}=b_{1}$. Thus, Player 1 considers that strategy in her game $G_{1}$ while preparing a best reply to that strategy. The next step is to extend rationalizability from strategic-form games to the games with unawareness.

**Definition** **4.** *An ESP ${\left\{{\left(\sigma \right)}_{v}\right\}}_{v\in V}$ in a game with unawareness is called extended rationalizable if for every v^v∈V strategy ${\left(\sigma_{v}\right)}_{v}$ is a best reply to (σ−v)v^v in the game Gv^v.*

Consider a strategic-form game with unawareness ${\left\{G_{v}\right\}}_{v\in V}$. For every relevant view v∈V, the relevant views as seen from *v* are defined to be Vv={v˜∈V:v^v˜∈V}. Then, the game with unawareness as seen from *v* is defined by {Gv^v˜}v˜∈Vv.

We are now in a position to define the Nash equilibrium in the strategic-form games with unawareness.

**Definition** **5.** *An ESP ${\left\{{\left(\sigma \right)}_{v}\right\}}_{v\in V}$ in a game with unawareness is called an extended Nash equilibrium (ENE) if it is rationalizable and for all $v,\bar{v}\in V$ such that {Gv^v˜}v˜∈Vv={Gv¯^v˜}v˜∈Vv¯ we have ${\left(\sigma \right)}_{v}={\left(\sigma \right)}_{\bar{v}}$.*

The first part of the definition (rationalizability) is similar to the standard Nash equilibrium, where it is required that each strategy in the equilibrium is a best reply to the other strategies of that profile. According to Definition 4, Player 2’s strategy ${\left(\sigma_{2}\right)}_{1}$ in the game of Player 1 has to be a best reply to Player 1’s strategy ${\left(\sigma_{1}\right)}_{12}$ in the game $G_{12}$. On the other hand, in contrast to the concept of Nash equilibrium, ${\left(\sigma_{1}\right)}_{12}$ does not have to a best reply to ${\left(\sigma_{2}\right)}_{1}$ but to strategy ${\left(\sigma_{2}\right)}_{121}$.

We saw in Section 2.1 that for v∈{1212,2121,…} we have $G_{v}=\Gamma_{C}$. It follows that {G1212^v}v∈V={G2121^v}v∈V={ΓC}. The second part of ENE implies that ${\left(\sigma \right)}_{21}={\left(\sigma \right)}_{121}$. The following proposition [17] shows that the notion of extended Nash equilibrium coincides with the standard one for strategic-form games when all views share the same perception of the game. It is therefore useful for determining ENE.

**Proposition** **1.** *Let G be a strategic-form game and ${\left\{G_{v}\right\}}_{v\in V}$ a strategic-form game with unawareness such that, for some v∈V, we have Gv^v¯=G for every $\bar{v}$ such that v^v¯∈V. Let σ be a strategy profile in G. Then,*

*1*. *σ is rationalizable for G if and only if ${\left(\sigma \right)}_{v}=\sigma$ is part of an extended rationalizable profile in ${\left\{G_{v}\right\}}_{v\in V}$.*
*2*. *σ is a Nash equilibrium for G if and only if ${\left(\sigma \right)}_{v}=\sigma$ is part of on an ENE for ${\left\{G_{v}\right\}}_{v\in V}$ and this ENE also satisfies (σ)v=(σ)v^v¯.*

**Remark** **1.** *We see from Equations (8) and (11) that, for every v^v^v¯∈V, a normal-form game Gv^v^v¯ and a strategy profile (σ)v^v^v¯ determine the games and profiles in the form Gv^v^…^v^v¯ and (σ)v^v^…^v^v¯, respectively, for example, $G_{121}$ determines $G_{122\ldots 21}$. Hence, in general, a game with unawareness ${\left\{G_{v}\right\}}_{v\in V}$ and an extended strategy profile ${\left\{{\left(\sigma \right)}_{v}\right\}}_{v\in V}$ are defined by ${\left\{G_{v}\right\}}_{v\in N\cup \{\emptyset \}}$ and ${\left\{{\left(\sigma \right)}_{v}\right\}}_{v\in N\cup \{\emptyset \}}$, where*

(12) $$N=\{v\in V|v=\left(i_{1},\ldots ,i_{n}\right)\;with\;i_{k}\neq i_{k+1}\;for\;all\;k\}.$$

*Then, we get ${\left\{G_{v}\right\}}_{v\in V}$ from ${\left\{G_{v}\right\}}_{v\in N\cup \{\emptyset \}}$ by setting $G_{\tilde{v}}=G_{v}$ for $v=(i_{1},\ldots ,i_{n})\in N$ and $\tilde{v}\;=\;\left(i_{1},\ldots ,i_{k},i_{k},i_{k+1},\ldots ,i_{n}\right)\in V$. For this reason, we often restrict ourselves to N∪{∅} throughout the paper.*

### 2.4. The Role of the Notion of Games with Unawareness in Quantum Game Theory

The notion of games with unawareness is designed to model game theory problems in which players’ perceptions of the game are restricted. It was shown in [17] that the novel structure extends the existing forms of games. Although it is possible to represent games with unawareness with the use of games with incomplete information (by using probability equal to 0 to the situations that a player is not aware of), the extended Nash equilibrium does not map to any known solution concept of incomplete information games. In particular, the set of extended Nash equilibria forms a strict subset of the Bayesian Nash equilibria.

Once we know that games with unawareness is a new game form, it is natural to study that type of games in the quantum domain. Having given a quantum game scheme that maps a classical game *G* to the quantum one Q(G), and having given a family of games $\left\{G_{v}\right\}$, a family of quantum games $Q(G_{v})$ can be constructed in a natural way. Then, we can study if, and to what extent, quantum strategies compensate restricted perception of players.

Besides $\left\{Q\left(G_{v}\right)\right\}$, the notion of game with unawareness allows one to expand the theory of quantum games by defining a family $\left\{Q{\left(G\right)}_{v}\right\}$, where each quantum game $Q{\left(G\right)}_{v}$ corresponds to a specific perception of players about the quantum game Q(G). In this case players may have restricted perception of how a quantum game is defined. A good example of that quantum game theory problem is the quantum PQ Penny Flip game [3]: one of the players is aware of having all the quantum strategies, the other player perceives two unitary strategies identified with the classical Penny Flip game. We provide a detail exposition of that problem in [18].

Another example of applying the notion of games with unawareness concerns the case when playing a quantum game is not common knowledge among the players. The quantum game is to be played with the aid of objects that behave according to the laws of quantum mechanics, in particular, the players may share an entangled two-qubit state on which they apply unitary strategies.

**Figure 1:**
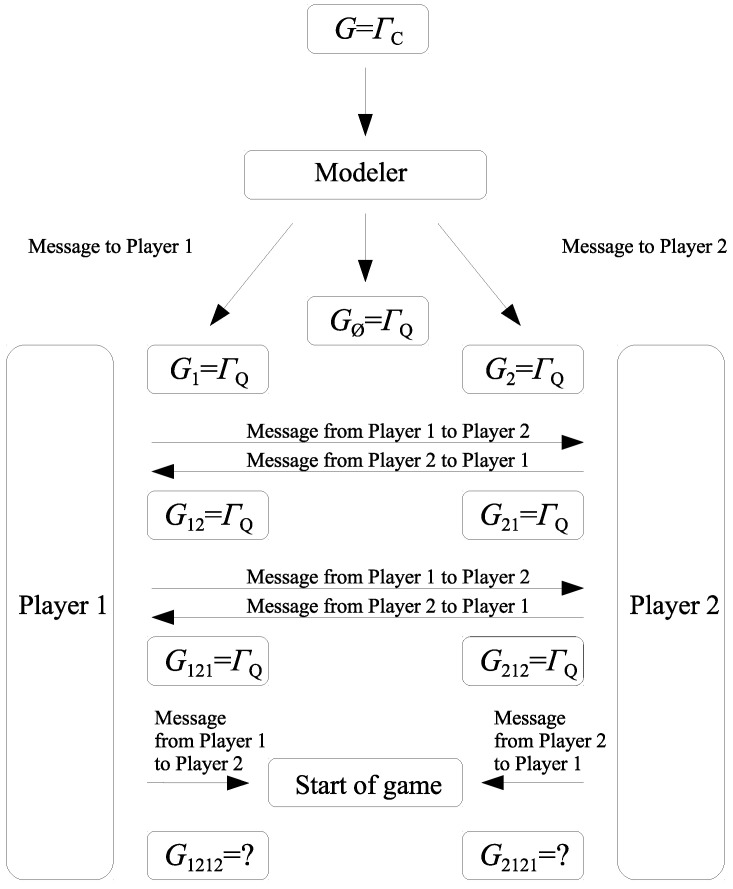
A possible scenario before a quantum game is played.

Under this scenario (see Figure 1), Players 1 and 2 can be far apart, and a third party, say a modeler, is to prepare the game. After the modeler prepares the quantum game based on its classical counterpart, she sends the message to Players 1 and 2 so that they know they are to play the quantum game rather than the classical one. When the players receive the message, each Player *i* perceives the game as being quantum, i.e., $G_{i}=\Gamma_{Q}$ for i=1,2. However, this fact is not common knowledge among Players 1 and 2. Recall that a fact is common knowledge among the players of a game if for any finite sequence of players $i_{1},i_{2},\ldots ,i_{k}$ Player $i_{1}$ knows that Player $i_{2}$ knows …that Player $i_{k}$ knows the fact. In our case, each of the players cannot be certain that the other player finds the quantum game (receives the message from the modeler) until he or she receives a confirmation from that player. According to the scheme in Figure 1, Players 1 and 2 send a message to each other about their own current state of knowledge. In this way, Player 1 receiving the message from Player 2 finds that Player 2 is considering the quantum game, i.e., $G_{12}=\Gamma_{Q}$. Similarly, Player 2 after receiving the message finds that Player 1 is also considering the quantum game, i.e., $G_{21}=\Gamma_{Q}$. The players continue to send messages to each other informing about their own knowledge. After receiving the message, Player 1 learns that Player 2 finds that Player 1 is considering the quantum game. As a result, Player 1 perceives the game $G_{121}$ as $\Gamma_{Q}$. In the same manner we can see that the game $G_{212}$ that Player 2 finds that Player 1 finds that Player 2 is considering is $\Gamma_{Q}$.

Two rounds of sending messages are still insufficient to say that $\Gamma_{Q}$ is common knowledge among the players. At the time the game starts, the games $G_{v}$ corresponding to higher order views v∈{1212,2121,12121,21212,…} are still unknown for the players, and either the classical game $\Gamma_{C}$ or the quantum game $\Gamma_{Q}$ may be associated with $G_{1212}$ and $G_{2121}$. As a result, the players face a game with unawareness described by a family of games ${\left\{G_{v}\right\}}_{v\in N_{0}\cup \left\{\emptyset \right\}}$ consisting of two types of games: $\Gamma_{Q}$ and $\Gamma_{C}$. An example of the game being in line with the scheme in Figure 1 is a family of games ${\left\{G_{v}\right\}}_{v\in N_{0}\cup \left\{\emptyset \right\}}$, where

(13) $$G_{v}=\left\{\begin{array}{cc} \Gamma_{Q} & \mathrm{if}\;v\in \{\emptyset ,1,2,12,21,121,212\},\\ \Gamma_{C} & \mathrm{otherwise}. \end{array}\right.$$

We show below that whether a quantum game $\Gamma_{Q}$ is common knowledge considerably affects the result of game.

## 3. Eisert–Wilkens–Lewenstein Scheme

We begin by recalling the Eisert–Wilkens–Lewenstein (EWL) scheme [20] in a general setting [21].

### 3.1. Construction

Let us consider a strategic-form game $G=(N,{\left(S_{i}\right)}_{i\in N},{\left(u_{i}\right)}_{i\in N})$ with N={1,…,n} and a two-element strategy set $S_{i}=\left\{s_{0}^{i},s_{1}^{i}\right\}$ for each Player i∈N. The generalized Eisert–Wilkens– Lewenstein approach to game *G* is a triple

(14) $$Q\left(G\right)=(N,{\left(D_{i}\right)}_{i\in N},{\left(u_{i}^{*}\right)}_{i\in N}),$$

where one has the following:

- $D_{i}$ is a set of unitary operators, $D_{i}\subset \mathrm{SU}\left(2\right)$. The commonly used parameterization for U∈SU(2) is given by
  (15) $$U\left(\theta ,\alpha ,\beta \right)=\left(\begin{array}{cc} e^{i\alpha }\cos\frac{\theta }{2} & ie^{i\beta }\sin\frac{\theta }{2}\\ ie^{-i\beta }\sin\frac{\theta }{2} & e^{-i\alpha }\cos\frac{\theta }{2} \end{array}\right),\theta \in \left[0,\pi \right],\alpha ,\beta \in \left[0,2\pi \right).$$
  Each set $D_{i}$ is assumed to include {U(θ,0,0)∣θ∈[0,π]}. Elements $U_{i}\in D_{i}$ play the role of strategies of Player *i*. Each Player *i*, by choosing $U_{i}\in D_{i}$, determines the final state |Ψ〉 according to the following formula:
  (16) $$|\Psi \rangle =J^{†}\left(\overset{n}{\underset{i=1}{⨂}}U_{i}\left(\theta_{i},\alpha_{i},\beta_{i}\right)\right)J{|0\rangle }^{⊗n},\;\mathrm{where}\;J=\frac{1}{\sqrt{2}}\left({𝟙}^{⊗n}+i\sigma_{x}^{⊗n}\right).$$
- $u_{i}^{*}$ is Player *i*’s payoff function. It is defined as the average value of the observable $M_{i}$,
  (17) $$M_{i}=\sum_{j_{1},\ldots ,j_{n}\in \left\{0,1\right\}}a_{j_{1},\ldots ,j_{n}}^{i}|j_{1},\ldots ,j_{n}\rangle \langle j_{1},\ldots ,j_{n}|.$$
  The numbers $a_{j_{1},\ldots ,j_{n}}^{i}$ are Player *i*’s payoffs in *G* such that $a_{j_{1},\ldots ,j_{n}}^{i}=u_{i}\left(s_{j_{1}}^{1},\ldots ,s_{j_{n}}^{n}\right)$. The function $u_{i}^{*}$ may be written as
  (18) $$u_{i}^{*}\left(\overset{n}{\underset{i=1}{⨂}}U_{i}\left(\theta_{i},\alpha_{i},\beta_{i}\right)\right)=\mathrm{tr}(|\Psi \rangle \langle \Psi |M_{i}).$$

### 3.2. Quantum Counterparts of Classical Strategies

Throughout this paper, we study the EWL quantum game in which some of the players are only aware of the classical strategies available to them and/or other players. We therefore need to determine precisely what quantum operations replicate the strategies in the classical game *G*. Based on Equations (15)–(17), a simple, yet tedious, calculation of Equation (18) reveals that the EWL scheme is equivalent to the game played classically if the players’ unitary strategies are restricted to the set {U(θ,0,0)∣θ∈[0,π]} (see, for example, [22] for the general payoff function of Equation (18) in the case n=2). Then, for any mixed strategy profile in *G*, there exists a payoff equivalent unitary profile ${⨂}_{i=1}^{n}U_{i}\left(\theta_{i},0,0\right)$ in Q(G) and vice versa. This equivalence of Player *i*’s mixed strategy $\left(p_{i},1-p_{i}\right)$ and a unitary matrix $U_{i}\left(\theta_{i},0,0\right)$ can be expressed by the following equation:

(19) $$\theta_{i}=2\arccos\sqrt{p},\;p\in \left[0,1\right].$$

A natural question that arises is whether the set {U(θ,0,0)∣θ∈[0,π]} can model the classical strategies when played against a strategy profile containing a full-parameter unitary strategy U(θ,α,β). The problem is not trivial, as we do not have a payoff function to compare with the one determined by a unitary strategy profile with at least one non-trivial strategy U(θ,α,β) in the EWL scheme. Therefore, to answer this question we need to appeal to properties of the payoff functions in *G* and Q(G). In most games, that we study in the classical game theory, we assume that the preference relations of the players are represented by a linear utility function $u_{i}$, or more precisely, they satisfy the von Neumann–Morgenstern axioms (see, for example, [23]). This implies, among other things, that Player *i*’s payoff in *G* resulting from playing a mixed strategy $\tau_{i}=\left(p_{i},1-p_{i}\right)$ (a probability distribution over $\left\{s_{0}^{i},s_{1}^{i}\right\}$) against $\tau_{-i}$ is the probability-weighted average of $u_{i}\left(\left(s_{0}^{i},\tau_{-i}\right)\right)$ and $u_{i}\left(\left(s_{1}^{i},\tau_{-i}\right)\right)$ according to $\left(p_{i},1-p_{i}\right)$,

(20) $$u_{i}\left(\left(p,1-p\right),\tau_{-i}\right)=pu_{i}\left(\left(s_{0}^{i},\tau_{-i}\right)\right)+\left(1-p\right)u_{i}\left(\left(s_{1}^{i},\tau_{-i}\right)\right).$$

Now, we can use Equation (20) as a criterion for checking whether the set {U(θ,0,0)∣θ∈[0,π]} models the set of mixed strategies {(p,1−p),p∈[0,1]}. We see from Equation (19) that $U_{i}\left(2\arccos\sqrt{p_{i}},0,0\right)$ is supposed to be equivalent to the mixed strategy (p,1−p) and, therefore, in particular, $U_{i}\left(0,0,0\right)=𝟙$ and $U_{i}\left(\pi ,0,0\right)=i\sigma_{x}$ are associated with the pure strategies $s_{0}^{i}=\left(1,0\right)$ and $s_{0}^{i}=\left(0,1\right)$, respectively. Consider the EWL scheme in Equations (15)–(18) with n=2 and the strategy sets $D_{1}=\left\{U_{1}\left(\theta_{1},0,0\right)|\theta_{1}\in \left[0,\pi \right]\right\}$ and $D_{2}=\mathrm{SU}\left(2\right)$. We first determine Player 1’s payoff corresponding to strategy profile $U_{1}\left(2\arccos\sqrt{p},0,0\right)⊗U_{2}\left(\pi /2,0,\pi /2\right)$. According to Equation (16), the final state |Ψ〉 is given by

(21) $$\begin{array}{cc} |\Psi \rangle & =J^{†}\left(U_{1}\left(2\arccos\sqrt{p},0,0\right)⊗U_{2}\left(\pi /2,0,\pi /2\right)\right)J|00\rangle \\ & =\left(\sqrt{p}+\sqrt{1-p}\right)|00\rangle -i\left(\sqrt{p}-\sqrt{1-p}\right)|10\rangle . \end{array}$$

Hence,

(22) $$\begin{array}{c} u_{1}^{*}\left(U_{1}\left(2\arccos\sqrt{p},0,0\right)⊗U_{2}\left(\pi /2,0,\pi /2\right)\right)\\ \;=\left(1/2+\sqrt{p}\sqrt{1-p}\right)a_{00}^{1}+\left(1/2-\sqrt{p}\sqrt{1-p}\right)a_{10}^{1}. \end{array}$$

From Equation (22) for p=1 and p=0, Player 1’s payoff resulting from playing 𝟙 and $i\sigma_{x}$ against $U_{2}\left(\pi /2,0,\pi /2\right))$ is $a_{00}^{1}/2+a_{10}^{1}/2$. We thus get

(23) $$\begin{array}{c} u_{1}\left(U_{1}\left(2\arccos\sqrt{p},0,0\right)⊗U_{2}\left(\pi /2,0,\pi /2\right)\right)\\ \;\neq pu_{1}\left(𝟙⊗U_{2}\left(\pi /2,0,\pi /2\right)\right)+\left(1-p\right)u_{1}\left(i\sigma_{x}⊗U_{2}\left(\pi /2,0,\pi /2\right)\right). \end{array}$$

In other words, Player 1’s payoff function with the set $\left\{U_{1}\left(2\arccos\sqrt{p},0,0\right)\right\}$ playing the role of the set of her mixed strategies (p,1−p) is not linear compared with the classical case. In particular, Player 1 by playing $U_{1}\left(\pi /2,0,0\right)$ (that is supposed to be equivalent to mixed strategy (1/2,1/2)) against Player 2’s strategy may not obtain the payoff that is the average of payoffs corresponding to her both pure strategies. A quick look at Equation (22) shows that the nonlinearity of the payoff function $u_{1}$ follows from the interference terms $\pm \sqrt{p}\sqrt{1-p}$. These terms are not part of the payoff function if the player’s classical mixed strategies (p,1−p) are modeled by quantum operation

(24) $$C_{p}\left(\rho \right)=p𝟙\rho 𝟙+\left(1-p\right)\sigma_{1}\rho \sigma_{1},$$

where ρ stands for a 2×2 density matrix. For this reason, we identify the classical mixed strategies in the EWL scheme with (24) throughout the paper.

### 3.3. Nash Equilibria in Eisert–Wilkens–Lewenstein-Type Game

Nash equilibrium is the primary solution concept for games in strategic form. It has become the most common tool in studying quantum games. In EWL-type games, a Nash equilibrium always exists. Moreover, part of Nash equilibria is independent of the payoff functions of the game. For example, in the EWL approach to a 2×2 game, playing U∈SU(2) at random with respect to the Haar measure on SU(2) is a Nash equilibrium strategy (see [21] for more details, and [24] for comprehensive analysis of Nash equilibria in quantum 2×2 games). The following proposition generalizes this statement to a set of players of arbitrary finite size. It is used repeatedly hereafter.

Let ρ denote a 2×2 density matrix. Define

(25) $$I\left(\rho \right)=\rho ,\;S\left(\rho \right)=\sum_{i=0}^{3}\sigma_{i}\rho \sigma_{i}^{†}/4,\;C\left(\rho \right)=\sum_{i=0}^{1}\sigma_{i}\rho \sigma_{i}^{†}/2,$$

where, for convenience, $\sigma_{i}$, i=1,2,3 stand for the Pauli matrices with $\sigma_{0}=𝟙$. Essential to the proof of the proposition is the following lemma:

**Lemma** **1.** *Let $|\Psi \rangle =\left({|0\rangle }^{⊗n}+i{|1\rangle }^{⊗n}\right)/\sqrt{2}$, n≥2 and $O_{j},j=1,\ldots ,n-1$, where*

(26) $$\left\{\begin{array}{cc} O_{j}\in \left\{S,C\right\} & if\;j\neq n-1,\\ O_{j}=S & if\;j=n-1. \end{array}\right.$$

*Then,*

(27) $$\left(I⊗\overset{n-1}{\underset{j=1}{⨂}}O_{j}\right)(|\Psi \rangle \langle \Psi |)=2^{-n}{𝟙}^{⊗n}.$$

**Proof.**  It is obvious that $\sigma_{1}$ (the Pauli matrix *X*) flips the state of a qubit of |Ψ〉 from |0〉 to |1〉. The matrix $\sigma_{2}$ (the Pauli matrix Y) flips both the state of a qubit (say, *k*-th one) and the phase of |Ψ〉. In other words, it changes the state |Ψ〉 into

(28) $$\left({|0\rangle }^{⊗k-1}{|1\rangle |0\rangle }^{⊗n-k}-{i|1\rangle }^{⊗k-1}{|0\rangle |1\rangle }^{⊗n-k}\right)/\sqrt{2}$$

(up to the global phase factor). The Pauli matrix $\sigma_{3}$, in turn, flips only the phase. It follows that

(29) $$\begin{array}{cc} \left(I^{⊗n-1}⊗S\right)(|\Psi \rangle \langle \Psi |) & =2^{-2}\sum_{i=0}^{3}\left({𝟙}^{⊗n-1}⊗\sigma_{i}\right)\left|\Psi \rangle \langle \Psi \right|\left({𝟙}^{⊗n-1}⊗\sigma_{i}^{†}\right) \end{array}$$

(30) $$\;\begin{array}{c} =2^{-2}\left((|0\rangle {\langle 0|)}^{⊗n-1}+(|1\rangle {\langle 1|)}^{⊗n-1}\right)⊗𝟙. \end{array}$$

Since

(31) $$O(|0\rangle \langle 0|)=O(|1\rangle \langle 1|)=\frac{1}{2}𝟙$$

for O∈{S,C}, we obtain

(32) $$\left({𝟙}^{⊗n-2}⊗O⊗S\right)(|\Psi \rangle \langle \Psi |)=2^{-3}\left({\left|0\rangle \langle 0\right|}^{⊗n-2}+{\left|1\rangle \langle 1\right|}^{⊗n-2}\right)⊗{𝟙}^{⊗2}.$$

Repeating the above reasoning n−3 times leads to our assertion. ☐

**Proposition** **2.** *Let $G=(N,{\left(S_{i}\right)}_{i\in N},{\left(u_{i}\right)}_{i\in N})$ be an n-person strategic-form game with $|S_{i}|=2$ for i=1,…,n, and let $Q\left(G\right)=(N,{\left(D_{i}\right)}_{i\in N},{\left(u_{i}^{*}\right)}_{i\in N})$ be the corresponding EWL quantum game. Then, a strategy vector ${\left(\tau_{i}^{*}\right)}_{i\in N}\in \prod_{i=1}^{n}\left\{S,C\right\}$ in which at least two components $\tau_{j_{1}}^{*}$ and $\tau_{j_{2}}^{*}$ are equal to S is a Nash equilibrium in Q(G).*

**Proof.**  Without loss of generality, we can assume that $\tau_{n}^{*}=S$ and examine Player 1’s strategy $\tau_{1}^{*}$. By Lemma 1, the final state $\rho_{f}$ resulting from playing $\left(𝟙,\tau_{2}^{*},\ldots ,\tau_{n}^{*}\right)$ is $2^{-n}{𝟙}^{⊗n}$. Hence,

(33) $$\rho_{f}^{\prime }=\left(U_{1}⊗{𝟙}^{⊗n-1}\right)\rho_{f}\left(U_{1}^{†}⊗{𝟙}^{⊗n-1}\right)=2^{-n}{𝟙}^{⊗n},$$

and $\rho_{f}^{\prime }$ is the final state corresponding to strategy profile $\left(U_{1},\tau_{2}^{*},\ldots ,\tau_{n}^{*}\right)$. Since Player 1’s unitary strategy $U_{1}$ does not affect the final state $\rho_{f}$ and consequently the payoff $\mathrm{tr}(\rho_{f}M_{i})$, the strategy $\tau_{1}^{*}\in \;\left\{S,C\right\}$ is a best reply to $\tau_{-1}^{*}=\left(\tau_{2}^{*},\ldots ,\tau_{n}^{*}\right)$. In general, by playing against the strategy combination $\tau_{-i}^{*}$ which contains at least one S, Player *i* is indifferent between all of her strategies, and hence ${\left(\tau_{i}^{*}\right)}_{i\in N}$ is a Nash equilibrium. ☐

It is worth noting that S is one of the multiplicity of quantum strategies for which the proposition holds, and is closely related to the notion of unitary 1-design. The following definition is taken from [25].

**Definition** **6.** *Let X be a finite subset of U(d), the group of d×d unitary matrices, and let w:X→R be a positive weight function (i.e., w>0, $\sum_{U\in X}w\left(U\right)=1$). Then, (X,w) is called a (weighted) unitary t-design if*

(34) $$\sum_{U\in X}w\left(U\right)U^{⊗t}⊗{\left(U^{†}\right)}^{⊗t}=\int_{U(d)}U^{⊗t}⊗{\left(U^{†}\right)}^{⊗t}dU,$$

*where dU is the Haar measure on U(d).*

The next proposition [26] shows a relationship between unitary 1-designs and orthonormal bases of $C^{d\times d}$.

**Proposition** **3.** *For any X⊂U(d), X is a unitary orthonormal basis of $C^{d\times d}$ if and only if (X,1/|X|) is a minimum unitary 1-design.*

Now, we can state a corollary of Proposition 2.

**Corollary** **1.** *Let (X,w) be a unitary 1-design. Proposition 2 holds if any strategy $\tau_{i}^{*}=S$ is replaced with a mixed strategy such that an operator U∈X is chosen with probability w(U).*

**Proof.**  Since ${\left\{\sigma_{i}\right\}}_{i=0}^{3}$ is a orthonormal basis of $C^{2\times 2}$, the pair $\left({\left\{\sigma_{i}\right\}}_{i=0}^{3},1/4\right)$ is a unitary 1-design. Hence, for a 2×2 density matrix ρ we have

(35) $$\sum_{i=0}^{3}\sigma_{i}\rho \sigma_{i}^{†}/4=\int_{U(2)}U\rho U^{†}dU=\sum_{U\in X}w\left(U\right)U\rho U^{†},$$

where (X,w) is a unitary 1-design. ☐

## 4. Quantum Games with Unawareness

In this section, we introduce the problem of unawareness in the EWL scheme. For convenience of exposition, we assume that the players are fully aware of the number of players in the game. Their perception, however, may be limited with respect to sets of strategies. Since proper subsets of SU(2) are called into question in the EWL-type quantum game scheme [22,27], and the set {U(θ,0,0)∣θ∈[0,π]} goes beyond the set of strategies in the classical game (see Section 3.2), we assume that the strategy set that each player perceives is either $\left\{𝟙,i\sigma_{x}\right\}$ or SU(2).

Clearly, the EWL-type quantum game $Q\left(G\right)=(N,{\left(D_{i}\right)}_{i\in N},{\left(u_{i}^{*}\right)}_{i\in N})$ is a strategic-form game. Thus, the concept of game with unawareness, as defined in Definition 1, applies to the quantum case. In view of the restrictions above, we consider a collection ${\left\{Q{\left(G\right)}_{v}\right\}}_{v\in V}$ of EWL-type games, where

1. the set of relevant views V is equal to the set of all potential views, i.e.,
  (36) $$V=\overset{\infty }{\underset{n=0}{⋃}}N^{\left(n\right)},\;\mathrm{where}\;N^{\left(n\right)}=\prod_{j=1}^{n}N,$$
2. for all v∈V
  (37) $${\left(D_{i}\right)}_{v}\in \left\{\left\{𝟙,i\sigma_{x}\right\},\mathrm{SU}\left(2\right)\right\},$$
  and for v^v˜∈V
  (38) if(Di)v={𝟙,iσx}then(Di)v^v˜=(Di)v,
3. for v^v^v^v˜∈V,
  (39) Gv^v^v^v˜=Gv^v^v˜
4. for i∈N, v∈V and $\tau \in {⨂}_{i=1}^{n}{\left(D_{i}\right)}_{v}$,
  (40) $${\left(u_{i}^{*}\right)}_{v}\left(\tau \right)={\left(u_{i}^{*}\right)}_{\emptyset }\left(\tau \right).$$

An extended Nash equilibrium is guaranteed to exist in a game with unawareness (see Proposition 3 in [17]). An interesting question that arises here is whether an analogous fact can be formulated in the quantum domain. With a little effort, we could show that ${\left\{Q{\left(G\right)}_{v}\right\}}_{v\in V}$ may fail to have an ENE under weaker assumptions of the sets ${\left(D_{i}\right)}_{v}$, for example, with (37) replaced by $\left\{𝟙,i\sigma_{x}\right\}\subset {\left(D_{i}\right)}_{v}\subset \mathrm{SU}\left(2\right)$. We can simply take ${\left\{Q{\left(G\right)}_{v}\right\}}_{v\in V}$ in which for some view $v=(i_{1},\ldots ,i_{n})$, the set ${\left(D_{i_{n}}\right)}_{v}$ is not compact. Hence, it might be the case that a best reply ${\left(\tau_{i_{n}}\right)}_{v}$ to (τ−in)v^in does not exists in game Q(G)v^in=Q(G)v and neither does an ENE. Interestingly, the existence of an ENE is guaranteed in ${\left\{Q{\left(G\right)}_{v}\right\}}_{v\in V}$ defined by Equations (36)–(40).

**Proposition** **4.** *A quantum game ${\left\{Q{\left(G\right)}_{v}\right\}}_{v\in V}$ with unawareness defined by Equations (36)–(40) has an ENE.*

**Proof.**  The first part of the proof is based on techniques originated in the work of [17]. Let ${\left\{Q{\left(G\right)}_{v}\right\}}_{v\in V}$ be the EWL-type game with unawareness. We define an auxiliary EWL game as follows:

Let *i* denote a player in $Q{\left(G\right)}_{\emptyset }$. The set of players in the auxiliary game is given by N (defined by (12)) The set of strategies $D_{v}$ for each Player $v=(i_{1},\ldots ,i_{n})\in N$ is given by

(41) $$D_{v}={\left(D_{i_{n}}\right)}_{v}\in \left\{\left\{𝟙,i\sigma_{x}\right\},\mathrm{SU}\left(2\right)\right\}.$$

Define the payoff function $U_{v}$ for each Player v∈N by

(42) $$U_{v}\left({\left({\left(a_{j}\right)}_{\tilde{v}}\right)}_{\tilde{v}\in N}\right)={\left(u_{i_{n}}^{*}\right)}_{\emptyset }\left({\left({\left(a_{j}\right)}_{v}\right)}_{j\in N}\right),$$

where ${\left(u_{i_{n}}^{*}\right)}_{\emptyset }\left({\left({\left(a_{j}\right)}_{v}\right)}_{j\in N}\right)$ is the payoff to Player $i_{n}$ in the EWL-type game $Q{\left(G\right)}_{v}$ that corresponds to the strategy profile ${\left({\left(a_{j}\right)}_{v}\right)}_{j\in N}$. Note that the payoff function $U_{v}$ depends only on a finite-dimensional strategy vector even though the game $\left(N,{\left(D_{v}\right)}_{v\in N},{\left(U_{v}\right)}_{v\in N}\right)$ has a countable number of players. To clarify (42), in case N={1,2}, the payoff function $U_{12}$ may be written as

$$\begin{array}{c} U_{12}\left({\left(a_{1}\right)}_{1},{\left(a_{2}\right)}_{2},{\left(a_{2}\right)}_{12},{\left(a_{1}\right)}_{21},{\left(a_{1}\right)}_{121},{\left(a_{2}\right)}_{212},\ldots \right)\\ \;\;\;={\left(u_{2}^{*}\right)}_{\emptyset }\left({\left(a_{1}\right)}_{12},{\left(a_{2}\right)}_{12}\right)={\left(u_{2}^{*}\right)}_{\emptyset }\left({\left(a_{1}\right)}_{121},{\left(a_{2}\right)}_{12}\right)=U_{12}\left({\left(a_{1}\right)}_{121},{\left(a_{2}\right)}_{12},\cdot \right), \end{array}$$

where the second to last equality follows from the definition of extended strategy profile. In general, the left-hand side of Equation (42) may be viewed as

(43) Uv(((aj)v˜)v˜∈N)=Uv((ain)v,((aj)v^j)j∈N,j≠in).

Let us assume that there are at least two views $v_{1}$ and $v_{2}$ from N for which $D_{v_{1}}=D_{v_{2}}=\mathrm{SU}\left(2\right)$. By Proposition 2, the game $\left(N,{\left(D_{v}\right)}_{v\in N},{\left(U_{v}\right)}_{v\in N}\right)$ has a Nash equilibrium ${\left({\left(a_{j}^{*}\right)}_{v}\right)}_{v\in N}$. The Nash equilibrium determines the extended Nash equilibrium $\left\{{\left({\left(a_{j}^{*}\right)}_{v}\right)}_{j\in N}\right){\}}_{v\in V}$ in the game ${\left\{Q{\left(G\right)}_{v}\right\}}_{v\in V}$, where the components ${\left(a_{j}^{*}\right)}_{\tilde{v}}$ for $\tilde{v}=\left(i_{1},\ldots ,i_{k},i_{k},i_{k+1},\ldots ,i_{n}\right)\in V\backslash N$ are given by ${\left(a_{j}^{*}\right)}_{\tilde{v}}={\left(a_{j}^{*}\right)}_{v}$ for $v=(i_{1},\ldots ,i_{n})\in V$. Indeed, by Equation (43), we have

(44) (uin∗)∅(((aj∗)v)j∈N)=Uv((aj∗)v˜)v˜∈N  ≥Uv((ain)v,((aj∗)v^j)j∈N,j≠in)=(uin∗)∅((ain)v,((aj∗)v)j∈N,j≠in)

for each $v=(i_{1},\ldots ,i_{n})\in N$ and each $a_{i_{n}}\in {\left(D_{i_{n}}\right)}_{v}$. Thus, ${\left(a_{i_{n}}\right)}_{v}$ is a best reply to (a−in)v=(a−in)v^in in game Q(G)v^in. By replacing *v* with v^j for $j\neq i_{n}$ in Equation (44), we obtain that ${\left(a_{j}^{*}\right)}_{v}$ is a best reply to (a−j∗)v^j in game Q(G)v^j. As a result, we have shown that $\left\{{\left({\left(a_{j}^{*}\right)}_{v}\right)}_{j\in N}\right){\}}_{v\in V}$ is rationalizable. Moreover, if $\tilde{v},\bar{v}\in V$ are the views such that {Q(G)v^v˜}v˜∈Vv={Q(G)v¯^v˜}v˜∈Vv¯ we deduce from (42) that Uv˜^j=Uv¯^j. Hence, the fact that ${\left(a_{j}^{*}\right)}_{\tilde{v}}$ is a best reply to (a−j∗)v˜^j in game Q(G)v˜^j is equivalent to stating that ${\left(a_{j}^{*}\right)}_{\bar{v}}$ is a best reply to (a−j∗)v¯^j in game Q(G)v¯^j. As a result, $\left\{{\left({\left(a_{j}^{*}\right)}_{v}\right)}_{j\in N}\right){\}}_{v\in V}$ is an extended Nash equilibrium.

We now turn to the case in which $D_{v}=\mathrm{SU}\left(2\right)$ for at most one v∈N. If $D_{v}=\left\{𝟙,i\sigma_{x}\right\}$ for all v∈N then ${\left\{Q{\left(G\right)}_{v}\right\}}_{v\in V}$ is equivalent to the classical game with unawareness, and by Proposition 3 in [17], a Nash equilibrium in the auxiliary game determines an extended Nash equilibrium ${\left\{{\left(\tau^{*}\right)}_{v}\right\}}_{v\in V}$, where each strategy in the profile ${\left(\tau^{*}\right)}_{v}={\left(\tau_{1}^{*},\ldots ,\tau_{n}^{*}\right)}_{v}$ may be viewed as a probability distribution over $\left\{𝟙,\sigma_{x}\right\}$. The only point remaining concerns $D_{v}=\mathrm{SU}\left(2\right)$ for exactly one v∈N. From Equation (38), it follows that *v* is the form v=i. Without restriction of generality we can assume that

(45) $${\left(D_{i}\right)}_{1}=\left\{\begin{array}{cc} \mathrm{SU}(2) & \mathrm{if}\;i=1,\\ \left\{𝟙,i\sigma_{x}\right\} & \mathrm{if}\;i\neq 1 \end{array}\right.\;\;\mathrm{and}\;\;{\left(D_{i}\right)}_{v}=\left\{𝟙,i\sigma_{x}\right\}\;\mathrm{for}\;v\in N\backslash \left\{1\right\}.$$

Let $U_{1}^{*}\in \mathrm{SU}\left(2\right)$ be a best reply to ${\left(\tau_{-1}^{*}\right)}_{1}$ in $Q{\left(G\right)}_{1}$. Such a strategy exists since Player 1’s payoff function $u_{1}^{*}\left(\cdot ,\tau_{-1}^{*}\right):\mathrm{SU}\left(2\right)\to R$ is a continuous function on the compact set SU(2). We construct an ENE in ${\left\{Q{\left(G\right)}_{v}\right\}}_{v\in V}$ by replacing ${\left(\tau_{1}^{*}\right)}_{1}$, ${\left(\tau_{1}^{*}\right)}_{11}$, …, ${\left(\tau_{1}^{*}\right)}_{11\ldots 1\ldots }$ in ${\left\{{\left(\tau^{*}\right)}_{v}\right\}}_{v\in V}$ with $U_{1}^{*}$ to obtain ${\left\{{\left(\sigma^{*}\right)}_{v}\right\}}_{v\in V}$, where

(46) $${\left(\sigma^{*}\right)}_{v}=\left\{\begin{array}{cc} {\left(\tau^{*}\right)}_{v} & \mathrm{if}\;v\in V\backslash \{\emptyset ,1,11,111,\ldots \},\\ \left(U_{1}^{*},{\left(\tau_{-1}^{*}\right)}_{1}\right) & \mathrm{if}\;v\in \{1,11,111,\ldots \},\\ \left(U_{1}^{*},{\left(\tau_{2}^{*}\right)}_{2},\ldots ,{\left(\tau_{n}^{*}\right)}_{n}\right) & \mathrm{if}\;v=\emptyset . \end{array}\right.$$

Since $U_{1}^{*}$ is a best reply to ${\left(\tau_{-1}^{*}\right)}_{1}$ and ${\left\{{\left(\tau^{*}\right)}_{v}\right\}}_{v\in V}$ is rationalizable, ${\left\{{\left(\sigma^{*}\right)}_{v}\right\}}_{v\in V}$ defined by Equation (46) is also rationalizable. Note that {Q(G)1^v˜}≠{Q(G)v^v˜} for v∈V\{∅,1,11,…}. Thus, the second condition of Definition 5 has no effect on ${\left(\sigma_{1}\right)}_{1}=U_{1}^{*}$. The family of profiles ${\left\{{\left(\sigma^{*}\right)}_{v}\right\}}_{v\in V}$ is therefore an ENE. ☐

Our first example shows that unawareness can be beneficial to the players.

**Example** **1.** *Consider a generalized form of the Prisoner’s Dilemma given by bimatrix*

(47) $$A:\begin{array}{cc} & \begin{array}{cc} C\; & \;D \end{array}\\ \begin{array}{c} C\\ D \end{array} & \left(\begin{array}{cc} \left(r,r\right) & \left(s,t\right)\\ \left(t,s\right) & \left(p,p\right) \end{array}\right) \end{array},t>r>p>s$$

*and its EWL counterpart Q(A) defined by Equations (15)–(18),*

(48) $$Q\left(A\right)=\{\left\{1,2\right\},\left\{\mathrm{SU}\left(2\right),\mathrm{SU}\left(2\right)\right\},\left\{u_{1}^{*},u_{2}^{*}\right\}\}.$$

*Recall that A has the unique Nash equilibrium (D,D) that leads to the payoff outcome (p,p). What makes the game in Equation (47) interesting is the fact that the players would get (r,r) if they both chose the strategy C. However, the strategy profile (C,C) is not stable as each player can deviate and even obtain t. On the other hand, playing Q(A) does not solve the dilemma as well. The game Q(A) has multiple Nash equilibria (see for example [24]) but no one leads to (r,r) in general.*

*Given Q(A), we form a game with unawareness ${\left\{Q{\left(A\right)}_{v}\right\}}_{v\in N\cup \{\emptyset \}}$, where*

(49) $$Q{\left(A\right)}_{v}=\left\{\left\{1,2\right\},\left\{{\left(D_{1}\right)}_{v},{\left(D_{2}\right)}_{v}\right\},\left\{u_{1}^{*},u_{2}^{*}\right\}\right\},$$

*and for v∈N∪{∅} (see (12) for the definition of N)*

(50) $${\left(D_{i}\right)}_{v}=\left\{\begin{array}{cc} \mathrm{SU}(2) & if\;v\in \{\emptyset ,1,2\},\\ \left\{𝟙,i\sigma_{x}\right\} & otherwise. \end{array}\right.$$

*The players are both aware of all the unitary strategies available in the game. However, each player perceives that the other player’s strategy set is $\left\{𝟙,i\sigma_{x}\right\}$. In other words, each player thinks that the other player is considering the classical game.*

*We now compute all the extended Nash equilibria of the game. We let ${\left\{{\left(\tau_{1}^{*},\tau_{2}^{*}\right)}_{v}\right\}}_{v\in V}$ stand for an ENE (it exists by Proposition 4). For v∈{1,2} and every v^v˜∈N game Q(A)v^v˜ is equivalent to A. Since strategies C and D in game A can be identified with 𝟙 and $i\sigma_{x}$, respectively, and the strategy profile (D,D) is a Nash equilibrium in A, it follows that $\left(i\sigma_{x},i\sigma_{x}\right)$ in a Nash equilibrium in Q(A)v^v˜. By Proposition 1, the strategy profile $\left(i\sigma_{x},i\sigma_{x}\right)$ is part of an ENE in ${\left\{Q{\left(A\right)}_{v}\right\}}_{v\in V}$, i.e.,*

(51) (τ1∗,τ2∗)v=(iσx,iσx)foreveryv^v˜∈N.

*We are left with the task of determining ${\left(\tau_{1}^{*},\tau_{2}^{*}\right)}_{v}$ for v∈{1,2}. We conclude from Equations (11) and (51) that*

(52) $${\left(\tau_{2}^{*}\right)}_{1}={\left(\tau_{2}^{*}\right)}_{12}=i\sigma_{x}={\left(\tau_{1}^{*}\right)}_{21}={\left(\tau_{1}^{*}\right)}_{2}.$$

*Then, it follows from Definition 4 that ${\left(\tau_{1}^{*}\right)}_{1}=U_{1}\left(\theta_{1}^{*},\alpha_{1}^{*},\beta_{i}^{*}\right)$ has to be a best reply to $i\sigma_{x}$ in the game $Q{\left(A\right)}_{1}$. Since for $U_{1}\in \mathrm{SU}\left(2\right)$ we have*

(53) $$\begin{array}{cc} u_{1}^{*}\left(U_{1}\left(\theta_{1},\alpha_{1},\beta_{i}\right),i\sigma_{x}\right) & =r\sin^{2}\beta_{1}\sin^{2}\left(\theta_{1}/2\right)+s\cos^{2}\alpha_{1}\cos^{2}\left(\theta_{1}/2\right)\\ & \;+t\sin^{2}\alpha_{1}\cos^{2}\left(\theta_{1}/2\right)+p\cos^{2}\beta_{1}\sin^{2}\left(\theta_{1}/2\right), \end{array}$$

*Player 1’s best reply $U_{1}\left(\theta_{1}^{*},\alpha_{1}^{*},\beta_{i}^{*}\right)$ to $i\sigma_{x}$ is defined by*

(54) $$\left(\theta_{1}^{*},\alpha_{1}^{*},\beta_{i}^{*}\right)\in \left\{0\right\}\times \left\{\pi /2,3\pi /2\right\}\times \left[0,2\pi \right).$$

*In other words, $U_{1}\left(\theta_{1}^{*},\alpha_{1}^{*},\beta_{i}^{*}\right)=\pm i\sigma_{z}$. The same conclusion can be drawn for ${\left(\tau_{1}^{*},\tau_{2}^{*}\right)}_{2}$. As a result, an ENE ${\left\{{\left(\tau^{*}\right)}_{v}\right\}}_{v\in N\cup \{\emptyset \}}$ in ${\left\{Q{\left(A\right)}_{v}\right\}}_{v\in N\cup \{\emptyset \}}$ is of the form*

(55) $${\left(\tau^{*}\right)}_{v}=\left\{\begin{array}{cc} \pm i\sigma_{z}⊗\pm i\sigma_{z} & if\;v=\emptyset ,\\ \pm i\sigma_{z}⊗i\sigma_{x} & if\;v=1,\\ i\sigma_{x}⊗\pm i\sigma_{z} & if\;v=2,\\ i\sigma_{x}⊗i\sigma_{x}. & otherwise \end{array}\right.$$

*Interestingly, the ENE yields each player a payoff of r,*

(56) $$u_{1(2)}^{*}\left(\pm i\sigma_{z}⊗\pm i\sigma_{z}\right)=r>p.$$

*We can see that suitably limited players’ perceptions of the game can increase the equilibrium payoffs.*

*It is worth noting that each player is always willing to play quantum strategies. In the game ${\left\{Q{\left(A\right)}_{v}\right\}}_{v\in N\cup \{\emptyset \}}$ given by Equation (50). each player finds that his opponent is playing the classical game. Thus, each player should assume that his opponent will play $i\sigma_{x}$–the unitary counterpart of the strategy D. The outcome $\left(i\sigma_{x},i\sigma_{x}\right)$ generates the payoff of p for each player. If a player has access to the unitary strategies, he will choose one given by Equation (54), i.e., $\pm i\sigma_{z}$, and will obtain the maximal possible payoff*

(57) $$u_{1}^{*}\left(\pm i\sigma_{z},i\sigma_{x}\right)=u_{2}^{*}\left(i\sigma_{x},\pm i\sigma_{z}\right)=t>p.$$

The next example shows that in some games with unawareness every strategy profile is a result of an ENE.

**Example** **2.** *Consider the EWL-type games $Q{\left(B\right)}_{v}=\left(N,{\left({\left(D_{i}\right)}_{v}\right)}_{i\in N},{\left(u_{i}^{*}\right)}_{i\in N}\right)$ with |N|≥3. For each view $v=(i_{1}i_{2},\ldots ,i_{m})\in N$, where $i_{k}\in N$, define ${\left(D_{i}\right)}_{v}$ as follows:*

(58) $${\left(D_{i}\right)}_{i_{1}}=\mathrm{SU}\left(2\right),\;{\left(D_{i}\right)}_{i_{1}i_{2},\ldots i_{m}}=\left\{\begin{array}{cc} \mathrm{SU}(2) & if\;i\in \{i_{1},i_{2}\},\\ \left\{𝟙,i\sigma_{x}\right\} & otherwise. \end{array}\right.$$

*Moreover, given $v=(i_{1},\ldots ,i_{m})$, $i_{k}\neq i_{k+1}$, we set ${\left(D_{i}\right)}_{\tilde{v}}={\left(D_{i}\right)}_{v}$ for $\tilde{v}=\left(i_{i},\ldots ,i_{k},i_{k},i_{k+1},\ldots ,i_{n}\right)$.*

*We identify an ENE $\left\{{\left(\tau^{*}\right)}_{v}\right\}$ that generates an arbitrary strategy profile in $Q{\left(B\right)}_{\emptyset }$. Consider first a strategy profile ${\left(\tau^{*}\right)}_{i_{1}i_{2}}$ of $\left\{{\left(\tau^{*}\right)}_{v}\right\}$ for $i_{1}\neq i_{2}$. Since Q(B)i1i2=Q(B)i1i2^v˜ for every $\tilde{v}$, by Proposition 1, ${\left(\tau^{*}\right)}_{i_{1}i_{2}}=\tau^{*}$, where $\tau^{*}$ is a Nash equilibrium in $Q{\left(B\right)}_{i_{1}i_{2}}$. We know from Equation (58) that the strategy sets of Player $i_{1}$ and $i_{2}$ in $Q{\left(B\right)}_{i_{1}i_{2}}$ are SU(2), whereas the strategy sets of the other players are $\left\{𝟙,i\sigma_{x}\right\}$. Applying Proposition 2, we conclude that the strategy profile $\left(\tau^{*}\right)$ in which players $i_{1}$ and $i_{2}$ play the quantum operation S and the other players play C is a Nash equilibrium in $Q{\left(B\right)}_{i_{1}i_{2}}$. By the above, for i∈N we obtain*

(59) $${\left(\tau_{i}^{*}\right)}_{i_{1}i_{2}}=\left\{\begin{array}{cc} S & if\;i\in \{i_{1},i_{2}\},\\ C & otherwise. \end{array}\right.$$

*Since two views $i_{1}i_{2}$, i1i2^v, v∈V share the same perception of the game, i.e., {Q(B)i1i2^v˜}v˜={Q(B)i1i2^v^v˜}v˜, we have (τ∗)i1i2^v=(τ∗)i1i2 according to Definition 5.*

*The task is now to find ${\left(\tau^{*}\right)}_{i_{1}}$. If $i\neq i_{1}$ then*

(60) $${\left(\tau_{i}^{*}\right)}_{i_{1}}={\left(\tau_{i}^{*}\right)}_{i_{1}i}=S,$$

*which follows from Equations (11) and (59). In the case $i=i_{1}$, we determine strategy ${\left(\tau_{i}^{*}\right)}_{i_{1}}$ by using the fact that ${\left(\tau_{i}^{*}\right)}_{i_{1}}$ is rationalizable, i.e., ${\left(\tau_{i}^{*}\right)}_{i_{1}}$ is a best reply to ${\left(\tau_{-i}^{*}\right)}_{i_{1}}$ in game $Q{\left(B\right)}_{i_{1}}$ (see Definition 4). We deduce from Equation (60) that each strategy of ${\left(\tau_{-i}^{*}\right)}_{i_{1}}$ is S. Then, by Lemma 1, Player i is indifferent between all of her strategies. We thus can set ${\left(\tau_{i}^{*}\right)}_{i_{1}}=U_{j}\left(\theta_{j},\alpha_{j},\beta_{j}\right)$. Since $i=i_{1}$, Equation (11) implies that ${\left(\tau_{i}^{*}\right)}_{i_{1}}={\left(\tau_{i}^{*}\right)}_{\emptyset }$. As a result, we obtain the following extended rationalizable strategy profile ${\left(\tau^{*}\right)}_{v}$ for every v∈N∪{∅}:*

(61) $${\left(\tau^{*}\right)}_{v}=\left\{\begin{array}{cc} \left(U_{1}\left(\theta_{1},\alpha_{1},\beta_{1}\right),\ldots ,U_{n}\left(\theta_{n},\alpha_{n},\beta_{n}\right)\right) & if\;v=\emptyset ,\\ \left(S_{1},\ldots ,S_{i-1},U_{i}\left(\theta_{i},\alpha_{i},\beta_{i}\right),S_{i+1},\ldots ,S_{n}\right) & if\;v=i,\\ \left(C_{1},\ldots ,C_{i_{1}-1},S_{i_{1}},C_{i_{1}+1},\ldots ,C_{i_{2}-1},S_{i_{2}},C_{i_{2}+1},\ldots C_{n}\right) & if\;v=i_{1}i_{2},\ldots ,i_{k}. \end{array}\right.$$

*The profiles ${\left(\tau^{*}\right)}_{v}$ we have just constructed constitute an ENE that implies the strategy profile ${⨂}_{i=1}^{n}U_{i}\left(\theta_{i},\alpha_{i},\beta_{i}\right)$ played in $Q{\left(B\right)}_{\emptyset }$.*

## 5. Conclusions

The results of this paper have substantially developed quantum game theory and enabled us to go beyond frequently investigated 2×2 games. Recent difficulties, caused by sophisticated methods, in finding rational vectors of strategies in quantum game may be reduced by introducing elements of unawareness of players. This follows from the fact that, in numerous cases of such games, the rational solution described by the notion of extended Nash equilibrium, as presented in the examples above, consists of pure strategies. We have shown that an extended Nash equilibrium always exists in the EWL-type quantum game. Moreover, limited perceptions of the players of how the other players view the game have a significant impact on an ENE. In many cases, the equilibrium result does not depend on the input classical game but merely on how the players’ unawareness is defined.

Our work provides new tools that might be utilized in allied sciences. The obtained results will enable one to study numerous economics problems formulated in terms of games with unawareness with the use of mathematical methods of quantum information. At the same time, these problems will enrich theory of quantum information through new examples that will show superiority of using quantum methods over methods of classical information theory.
